# Diacerein-loaded surface modified iron oxide microparticles (SMIOMPs): an emerging magnetic system for management of osteoarthritis via intra-articular injection

**DOI:** 10.3389/fbioe.2024.1439085

**Published:** 2024-10-28

**Authors:** Nouran Abdelmageed Ali, Nadia M. Morsi, Shaimaa M. Badr-Eldin, Rehab N. Shamma

**Affiliations:** ^1^ Department of Pharmaceutics and Industrial Pharmacy, Faculty of Pharmacy, Cairo university, Jeddah, Egypt; ^2^ Department of Pharmaceutics, Faculty of Pharmacy, King Abdulaziz University, Jeddah, Saudi Arabia; ^3^ Center of Excellence for Drug Research and Pharmaceutical Industries, King Abdulaziz University, Jeddah, Saudi Arabia

**Keywords:** diacerein, iron oxide microparticles, osteoarthritis, intra-articular injection, rat model, factorial design

## Abstract

**Introduction:**

Osteoarthritis (OA) is regarded as one of the most prevealent irreversible joint degenerative disorder worldwide. Recently, considerable interest in utilizing intra-articular (IA) injections for managing OA has been raised.

**Methods:**

In this study, IA injectable surface modified iron oxide microparticles (SMIOMPs) loaded with Diacerein (DCN) were developed. The effects of formulation parameters on particle size, entrapment efficiency, and zeta potential were explored using factorial design. The optimized formulation was characterized regarding morphology and in vitro release. Differential scanning calorimetry (DSC) and Fourier-transform infrared spectroscopy (FTIR) were done to assess interactions. Further, sterilization and *in vivo* performance in rats with induced arthritis has been performed for the optimized formulation.

**Results and Discussion:**

The selected optimized system included 2M FeCL3 and 1% chitosan as a surface modifier achieved high drug entrapment of 85.25% with a PS of 1.54 µm and sustained DCN release. Morphological examination of the optimized formulation revealed spherical particles with chitosan coat. DSC and FTIR results indicated the absence of undesired interactions between DCN and the used components. No significant change in the measured parameters was observed following sterilization using gamma radiation. *In vivo* assessment revealed superior performance for the optimized formulation in reducing cartilage inflammation and degradation. Plasma levels of tumor necrosis factor α and Interleukin-1 beta, as well as knee diameter, were significantly reduced in the treated groups compared to the untreated ones.

**Conclusion:**

Overall, the results suggest that the proposed DCN-loaded SMIOMPs represent a promising advancement in the arena of cartilage regeneration.

## 1 Introduction

Osteoarthritis (OA) is regarded as one of the most dominant, possibly an irreversible joint degenerative disorder globally. The worldwide incidence of knee OA was 16% in population aged ≥ 15 years and 22.9% in those aged ≥ 40 years. Moreover, in 2020 about 654.1 million aged ≥40 years individuals over the world had knee OA (Neogi, 2013; Cui et al., 2020a).

Tissue engineering, an innovative approach merging engineering and life sciences, aims to create biological substitutes for repairing and regenerating damaged tissues (Howard et al., 2008). Magnetic hydrogels, formulated via iron oxide particles combined with diverse biopolymers, are gaining prominence in biomedical tissue engineering due to their biocompatibility, well-defined structures, and responsiveness to magnetic fields (Liu et al., 2020). Other smart biomaterials, including scaffolds and biofilms, which are activated by external stimuli, offer great potential in the biomedical field; however, they have pitfalls including delayed response and less defined architectures compared to biomaterials responding to magnetic stimuli (Cui et al., 2020b; Yang et al., 2020b).

Biomaterials are allocated based on different parameters including origin, composition, and biodegradability. Based on chemical composition, biomaterials can be categorized into ceramics, polymers, and composites (Veeman et al., 2021). In tissue engineering, biopolymers like chitosan and collagen are extensively used, particularly for cartilage regeneration. Chitosan, derived from deacetylated chitin, is a highly versatile natural polymer known for its biocompatibility, biodegradability, cationic character, stability, non-toxicity, and ability to be sterilized. Chitosan has different applications in drug delivery, tissue engineering, and wound healing. Its mucoadhesive characteristics allow it to serve as a scaffold anchorage to existing tissue (Comblain et al., 2017). Chitosan or modified chitosan enriched cartilage matrix compounds showed reduced inflammation and catabolic mediator’s levels by the chondrocytes in the *in-vitro* studies (Oprenyeszk et al., 2015).

Evolution of novel drug-delivery systems with high efficacy, low cytotoxicity and controlled drug release has recently gained much attention. Use of magnetic field and/or magnetic nanoparticles become one of the potential approaches to achieve such aims. Static magnetic field (SMF) is a is a persistent field that outlines the magnetic effect of electrical currents and magnetized materials on living matter. Widely applied in medicine, SMF is known to enhance wound healing and bone regeneration, particularly in physiotherapy for bone disorders such as osteoarthritis (Marycz et al., 2018). Magnetic discs, capable of generating SMF both *in vitro* and *in vivo*, provide practical applications in this field. The utilization of magnetic fields in clinical applications has attracted attention, particularly in magnetically guided delivery of biomolecules for tissue engineering, magnetic resonance imaging, and cancer therapy. However, ongoing debates surround the biological responses and potential adverse effects of SMF exposure (Marycz et al., 2018). Previous studies have demonstrated the efficacy of local SMF stimulation in treating pain, promoting nerve regeneration, and improving blood flow (Gmitrov et al., 2002; Veliks et al., 2004; Kanai et al., 2012) Furthermore, SMF has shown promise in the field of drug delivery. Its ability to concentrate magnetic particles and facilitate the targeted delivery of drug molecules underscores its potential significance in advancing medical treatments (Nguyen et al., 2021).

Magnetic systems have been recently regarded as a promising approach for the treatment of osteoarthritis, leveraging their ability to provide targeted therapy with minimal invasiveness. Magnetic particles are often employed because of their capacity to be directed by external magnetic fields, allowing precise localization to the damaged cartilage. Once localized, the particles can deliver therapeutic agents or induce hyperthermia, thereby promoting tissue repair and reducing pain and inflammation through cellular and molecular signaling pathways. Studies have shown that magnetic particles can effectively promote drug delivery and improve the bioavailability and retention of treatments in the affected joints, offering a novel pathway for osteoarthritis management (Wang et al., 2019; Kianfar, 2021; Materón et al., 2021). Research also suggests that MNP-based systems could be combined with tissue engineering strategies, such as scaffolds, to further improve cartilage repair in osteoarthritic joints (Fan et al., 2020; Dasari et al., 2022). The minimally invasive nature of magnetic systems, combined with their ability to limit off-target effects and reduce systemic toxicity, makes them a highly attractive therapeutic option for osteoarthritis safer alternative to conventional therapies (Liu et al., 2021; Materón et al., 2021).

Naturally available iron oxides, such as magnetite, maghemite, and hematite exhibit unique properties. Magnetite, the most widely used form, displays inter-valence charge transfer between Fe^2+^ and Fe^3+^, resulting in absorption in the ultraviolet-visible and infrared spectral regions and a black appearance. Magnetic iron oxide particles, notably Fe3O4 and γ-Fe2O3, are valuable in scientific and technological applications due to their minimal toxicity, superparamagnetic properties, and simple separation techniques. Their significance is particularly evident in biomedical applications, including drug delivery, diagnostic imaging with magnetic resonance, and thermal therapy (Ali et al., 2016).

The preparation of iron oxide particles involves methods such as thermal decomposition and co-precipitation. Co-precipitation is gaining attention due to its large yield. Chemical coprecipitation is dependent on several factors including ionic strength, iron salt type, and pH. It could be achieved either by addition of a base to an aqueous solution containing Fe^2+^ and Fe^3+^ ions or partial oxidation of ferrous hydroxide utilizing different oxidizing factors (Wu et al., 2011). Prepared iron oxide particles often lack stability and require stabilization using low molecular weight agents like surfactants or functionalized polymers. Surface modification for preserving the particles stability involves either physical or chemical approaches. Chemical approach includes surface-controlled polymerization or grafting methods, while physical encapsulation modifies particles through self-assembly, surfactant adsorption, or layer-by-layer electrostatic adsorption (Jamshaid et al., 2016).

Magnetic hydrogels consist of a hydrogel matrix combined with a magnetically surface-modified biopolymer component. Recently, there has been a prevalent trend of surface modifying iron oxide-based magnetic particles with polymer matrices, to create a magnetically responsive hydrogels for tissue engineering, like γ-Fe2O3, Fe3O4, and cobalt ferrite nanoparticles (Zhang and Song, 2016; Rose et al., 2017). Several studies have successfully reported the development of chitosan and collagen coated magnetic iron oxide particles dispersed into hydrogel for various biomedical applications (Zhang et al., 2014; Tóth et al., 2015; Jauch et al., 2020).

In this work, we aimed to prepare iron oxide magnetic microparticles followed by surface modification with hydrogel forming biopolymers like chitosan oligosaccharide lactate (CS) or hydrolyzed collagen (CO) owing to their beneficial effect in the OA management regarding their lubricant and tissue regeneration ability. The developed surface modified iron oxide magnetic microparticles (SMIOMPs) were loaded with DCN to be delivered via IA injection. The prepared SMIOMPs characteristics were optimized for minimized particle size, maximized entrapment efficiency and absolute zeta potential. The optimal DCN-loaded SMIOMPs morphology was visualized using transmission electron microscopy. Further, *in vivo* performance of the optimized DCN-loaded system was assessed in rat model with induced arthritis. Reduction in cartilage inflammation and degradation was evaluated. In addition, inflammatory condition was evaluated via measuring plasma levels of tumor necrosis factor α and Interleukin-1 beta, as well as knee diameter.

## 2 Materials and methods

### 2.1 Materials

Diacerein (DCN) was kindly gifted from EVA Pharmaceutical Industries (Cairo, Egypt). Ferric chloride (FeCl_3_), and Ferrous sulphate (FeSO_4_) was procured from Chemajet chemical company, Alexandria, Egypt. Cellulose dialysis membrane (molecular weight cut-off = 14,000 Da), Hydrolyzed collagen (CO), Chitosan oligosaccharide lactate (CS), Freund’s adjuvant, Ovalbumin, Hematoxylin and Eosin were obtained from Sigma Aldrich Co., St. Louis, MO, United States. Cal-Ex II Decalcifier, was obtained from Fisher Scientific, Leicestershire, United Kingdom ELISA kit was obtained from Glory Science Co, Ltd., Del Rio, TX, United States. All other chemicals and reagents were of analytical grade.

### 2.2 Preparation of DCN-loaded surface modified iron oxide magnetic microparticles (SMIOMPs)

Iron oxide particles were prepared via the co-precipitation technique (Lagrow et al., 2019), using ferric sulphate (FeSO_4_) and ferric chloride (FeCL_3_) solutions at the molar ratio of 1:1 or 1:2. Equal volumes of each solution were mixed on a magnetic stirrer (500 rpm) for 20 min at 55°C. The pH of mixture was increased via the addition of 4 mL basic solution (33% w/v ammonia solution) to allow the chemical precipitation of iron oxide particles. The prepared iron oxide particles were then collected by the aid of neodymium magnet. Washing of the collected particles was performed four times with deionized water to remove excess base, leaving a black precipitate of iron oxide microparticles; IOMPs (Sulistyaningsih et al., 2017). The prepared IOMPs were then mixed with 10 mL of either chitosan (CS) or hydrolyzed collagen (CO) solution at the specified concentration for surface modification. The dispersion was further stirred constantly for a further 12 h at 500 rpm to ensure surface modification. Finally, DCN (20 mg) was added into 10 mL of the resulted dispersion and stirring was continued for further 15 min to form DCN-loaded SMIOMPs.

### 2.3 *In vitro* characterization of the prepared DCN-Loaded IOMPs and SMIOMPs

#### 2.3.1 DCN entrapment efficiency determination

Efficiency of DCN entrapment (EE%) was done indirectly via measuring the unentrapped (free) DCN. Magnetic separation of 1 mL sample of the magnetic particles using a neodymium magnet was done for free DCN from the SMIOMPs. The resulted supernatant was withdrawn and the concentration of free DCN was measured by UV spectrophotometry at λ_max_ 258 nm after appropriate dilution (Eladawy et al., 2021). Drug EE% was determined according to the following equation:

$$\text{EE}\%=\frac{\text{Total}\;\text{DCN}-\text{Free}\;\text{DCN}}{\text{Total}\;\text{DCN}}\times 100$$

Each measurement was repeated thrice, and the results were presented as mean ± SD.

#### 2.3.2 Size and zeta potential assessment

Estimation of the average particle size (PS), polydispersity index (PDI), and zeta potential (ZP) of the prepared SMIOMPs was done using dynamic light scattering technique by Zetasizer (Maguire et al., 2018). Each system was examined after being diluted with distilled water to an appropriate dilution that could provide an appropriate scattering intensity allowing for proper size assessment. To observe the electrophoretic mobility of the particles within the electric field, ZP measurements were conducted using the same apparatus. Each sample was examined in triplicate, and the average value was displayed in the findings.

### 2.4 Formulation and optimization of DCN-loaded SMIOMPs using full factorial design

Various systems of DCN-loaded SMIOMPs were prepared according to 2^3^ full factorial design. Each variable was used at two levels as follows: molar concentration of FeCl_3_ (0.1 and 0.2 M), type of surface modifier (CO and CS), and concentration of surface modifier (0.5, 1%). Eight formulations were developed as per the experimental design employed. The composition of various systems formulated is represented in Table 1. The effects of the studied variables on the EE%, PS, PDI, and absolute ZP of the prepared formulations were investigated. Iron oxide microparticles were prepared without surface modification using 0.1 and 0.2 M FeCl_3_ were prepared for comparison. Design expert^®^ 13.0 was applied to analyze the responses and outline the optimal formulation with least PS and highest EE and absolute zeta potential.

**TABLE 1:** Composition and *in vitro* characterization of DCN-loaded SMIOMPs.

Formulation	FeCl_3_ molar concentration (M)	Surface modifier type	Surface modifier concentration (%)	Entrapment efficiency (%) ± SD^a^	Particle size (µm) ± SD^a^	Polydispersity index ± SD^a^	Zeta potential (mV) ± SD^a^
S1	1.0	-	-	43.10 ± 0.03	2.24 ± 0.02	0.350 ± 0.001	3.00 ± 0.0
S2	2.0	-	-	80.50 ± 0.02	1.54 ± 0.06	0.520 ± 0.003	4.70 ± 0.4
F1	1.0	CS	0.5	80.17 ± 0.025	3.12 ± 0.13	0.356 ± 0.003	26.00 ± 0.7
F2	1.0	CS	1.0	83.37 ± 0.125	2.40 ± 0.08	0.444 ± 0.004	35.95 ± 0.5
F3	1.0	CO	0.5	22.65 ± 0.150	5.15 ± 0.18	0.796 ± 0.002	−10.40 ± 0.4
F4	1.0	CO	1.0	25.05 ± 0.05	2.70 ± 0.02	0.766 ± 0.002	−34.80 ± 0.1
F5	2.0	CS	0.5	83.15 ± 0.15	3.02 ± 024	0.638 ± 0.003	28.40 ± 0.6
F6	2.0	CS	1.0	85.25 ± 0.25	1.56 ± 0.07	0.518 ± 0.001	41.35 ± 0.7
F7	2.0	CO	0.5	48.10 ± 0.1	3.83 ± 0.08	0.350 ± 0.003	−16.95 ± 0.1
F8	2.0	CO	1.0	73.05 ± 0.05	3.05 ± 0.09	0.799 ± 0.002	−51.20 ± 0.8

^a^ Data are mean values (n = 3) ± SD.

All formulations were loaded with 2 mg/mL diacerein.

### 2.5 Characterization of the optimal DCN-loaded SMIOMPs

#### 2.5.1 *In vitro* release

The release of DCN from the selected DCN-loaded SMIOMPs compared to the corresponding formulation without surface modification (DCN-loaded IOMPs) and DCN aqueous dispersion was studied using dialysis bag diffusion technique (Elsherif et al., 2021). The dialysis membrane was soaked in phosphate buffer saline (PBS, pH = 7.4) overnight before the experiment. One mL of each of the tested formulation and the drug dispersion (each equivalent to 2 mg DCN) was put in a dialysis bag. Immersing the dialysis bag in a bottle with 20 mL of PBS (pH = 7.4) was done in a shaking water bath operated at 50 strokes per minute. The water bath was thermostatically controlled at 37°C ± 0.5 °C. At predetermined intervals of 2, 4, 6, 8, 24, and 48 h, samples of the release media were taken out and replaced with an equivalent amount of release medium. The samples were subjected to spectrophotometric analysis at λ_max_ 258 nm and the amount released was then computed with reference to the constructed calibration curve. The study was done in triplicate and the results were displayed as mean ± SD.

#### 2.5.2 Shape and morphology assessment

The morphology of the selected DCN-loaded SMIOMPs and the corresponding DCN-loaded IOMPs without surface modification was investigated using transmission electron microscopy (TEM). One drop of each undiluted sample was mounted onto a carbon-coated copper grid. A drop of 1% (w/v) phosphotungstic acid was then applied to stain the samples, which were then allowed to dry in the air at ambient temperature before being examined at an 80 kV accelerating voltage (El Zaafarany et al., 2010).

#### 2.5.3 Fourier transform infrared (FTIR) spectroscopy

FTIR spectra, ranging from 4,000 to 400 cm^−1^, of DCN, FeCl3, FeSO4, CS, and the optimal freeze-dried DCN-loaded and blank SMIOMPs were recorded using the KBr disc technique (Nafee et al., 2018). The recorded spectra were examined for the presence of any chemical interaction.

#### 2.5.4 Thermal behavior analysis

The thermal analysis of DCN, FeCl_3_, FeSO_4_, CS, and the optimal freeze dried DCN-loaded and the corresponding blank SMIOMPs was performed via differential scanning calorimetry (DSC). Each sample was placed in a flat-bottom aluminum pan and heated steadily in nitrogen atmosphere (Rostamnezhad et al., 2023).

#### 2.5.5 pH evaluation

pH values for the prepared system were assessed utilizing a pH meter (Jenway 3505, Bibby Scientific) as previously reported (Abdelhakeem et al., 2021).

### 2.6 Sterilization of DCN-loaded SMIOMPs

Gamma irradiation (^60^Co irradiator) at a low dosage of 10 kGy (1.19 kGy/h for 8 h) was used to sterilize the optimized DCN-loaded SMIOMPs (Da Costa Martins et al., 2009). The gamma-sterilized formulation was re-evaluated for PS, ZP, and EE%.

### 2.7 *In-vivo* study

Experimental procedures followed the guide of the National Institute of Health for care and use of Laboratory animals (NIH Publications No.8023, revised 1978), and approved by the Research Ethics Committee of Faculty of Pharmacy Cairo University (PI 2847).

For this investigation, 42 male Albino rats weighing between 250 and 300 g were housed in an air-conditioned room at 25°C ± 0.5°C, with free access to water and a standard pellet diet. The animals were accommodated in cages with 12-h day and night cycles. 0.5 mL of ovalbumin (5 mg/mL) in complete Freund’s adjuvant was injected into rats knee joints two times for 2 weeks (at days 1 and 7) to induce OA (Kamel et al., 2016).

Rats were randomized into five groups; with each group comprising six rats.

• Group 1 (negative control) is a group of rats with healthy knee joints with no induction of OA.
• Group 2 (positive control) is a group of rats without any treatment.
• Group 3 is a group of rats that received 0.5 mL of blank SMIOMPs via intra-articular injection.
• Group 4 is a group of rats that received 0.5 mL of DCN aqueous dispersion (2 mg/mL) via intra-articular injection.
• Group 5 is a group of rats IA that received 0.5 mL of the optimized DCN-loaded SMIOMPs (F6) via intra-articular injection.

Among all groups the left knee joint was used as control and kept free of treatment, the right knee in groups 6 and 7 only were wrapped with a neodymium magnet (Allen et al., 2010). Blood samples were withdrawn from the retro-orbital plexus of the rats at the 28^th^ day and kept in tube with a separator of gel barrier serum. Centrifugation of the samples was done at room temperature for 15 min at a speed of 5,000 rpm for obtaining serum. Finally, the serum samples were kept at 0 °C till further analysis. For rats’ knees diameter determination, the antero-posterior diameters were measured at 7-day interval time points from day 1 to day 28 to evaluate the swelling of knee joints (Hartmann et al., 2018). The knee diameter baseline value was recorded just prior to adjuvant injection in each rat. Tumor necrosis factor α (TNF-α) and Interleukin-1 beta (IL-1b) serum levels were determined using ELISA kit as per the manufacturer’s guidelines (Yin et al., 2018).

### 2.8 Histopathologic studies

Sedation and euthanizing of the rats were achieved via sodium pentobarbital (50–80 mg/kg) intravenous delivery on the 28^th^ day, then both knees were isolated. The knee joints samples were immersed in 10% neutral buffered formalin for 48h, then decalcification of the samples was done using Cal-Ex II Decalcifier for 18 days. Samples were processed by serial dilutions of ethanol, followed by clearing in xylene before being submerged in paraplast tissue embedding medium. Using a rotatory microtome, tissue sections of approximately 5 µm were cut. After being cut at mid joint level, serial sagittal tissue sections were mounted onto glass slides for staining with hematoxylin and eosin (H&E) to be inspected for histological alterations. Ultimately, a full HD microscopic imaging system was used for samples imaging. The morphological assessment of tibiofemoral articular cartilage was performed using the Modified Mankin scoring system (Pauli et al., 2012; El-Gogary et al., 2020), with 0 corresponds to normal cartilage and 14 corresponds to the highest score of OA.

## 3 Results and discussion

### 3.1 *In vitro* characterization of DCN-loaded IOMPs and SMIOMPs

EE%, PS, PDI, and ZP of different DCN-loaded IOMPs and SMIOMPs systems are compiled in Table 1. Non-surface modified IOMPs containing 2 M FeCL_3_ (S2) could achieve better EE%, lower PS and higher absolute ZP than IOMPs with 1M FeCL_3_ (S1). This might be attributed to the initial Fe^2+^/Fe^3+^ molar ratio of 0.5 required for the formation of Fe_3_O_4_ (Mizutani et al., 2008). Increasing this ratio altered the particles properties, where absolute ZP become lower indicating particles instability, thus particles tend to agglomerate resulting in higher PS (Pochapski et al., 2021). All formulations showed suitable pH values for IA injection (pH 6.2–7.2) (Goldie and Nachemson, 1970; Petit et al., 2015; Küçüktürkmen et al., 2017).

Results revealed that surface modification of IOMPs resulted in a rise in the ZP of particles which indicates enhanced stability and lower particles aggregation tendency. DCN-loaded SMIOMPs containing CS had positive ZP values, while those containing CO had negative ZP values. This could be attributed to the positive charge of CS and the negative charge of CO, which serve as coating material (Cheung et al., 2015; Yang et al., 2020a).

SMIOMPs systems containing CS showed reasonable EE% (from 80% to 85%), while those containing CO showed lower EE% (from 22% to 73%). This might be credited to the acidic nature of DCN. The drug possesses an ionizable carboxylic group, which could possibly ionize and gain a negative charge in the neutral and alkaline pH. Since CO is also negatively charged, this could lead to their repulsion and decreased EE% (Aziz et al., 2018). This could be confirmed by the increased absolute ZP values and negative charge intensity upon increasing CO concentration. Additionally, the prepared systems showed PS ranging from 1.54 to 5.15 µm with appropriate size distribution (PDI from 0.03 to 0.79) and ZP from −51 to +41 mv.

### 3.2 Experimental design analysis

#### 3.2.1 Impact of variables on EE%

ANOVA analysis of the EE% results showed that all the investigated variables (molar concentration of FeCL_3_, concentration of surface modifier and type of surface modifier) had significant effect at *p* ≤ 0.05. The main effects and the interactions between the tested parameters of DCN-loaded SMIOMPs on EE% are illustrated Supplementary Figure S1.

Increasing the molar concentration of FeCL_3_ increases the EE % of DCN in the prepared SMIOMPs (*p* < 0.0001). It is worthy to note that the ratio of Fe^2+^: Fe^3+^ ratio (1:2) was found to be the optimum ratio at which the co-precipitation reaction occurs for preparation of iron oxide particles (Alibeigi and Vaezi, 2008). A previous study by Gupta and Gupta (2005) reported that to avoid the formation of oxide mixtures, the theoretical molar ratio of Fe^2+^ and Fe^3+^ salts should be 1:2 for complete synthesis of Fe_3_O_4._ Hence, more stable particles allow higher EE% of DCN.

Regarding the surface modifier type, it was observed that CS modified SMIOMPs had higher DCN EE % than CO surface modified ones (*p* < 0.0001). This might be attributed to higher mechanical strength and higher viscosity of CS solution than CO solution (León-López et al., 2019; Prasanna Mallick et al., 2021), which lead to higher DCN EE % as it prevents drug leakage from the particles. Also, CS provides different functional groups including reactive hydroxyl and amino groups which may bind to the drug molecules leading to improved drug entrapment (Taherian et al., 2021).

Increasing the surface modifier concentration from 0.5% to 1% increased the DCN EE% (*p* < 0.0001). This might be attributed to increase in the viscosity of the media upon increasing the surface modifier concentration, which prevents the migration of DCN to the outer phase (Dhakar et al., 2012). Similar observations were reported by Huang et al. (2018) in their study on preparation of vancomycin-loaded silica xerogel/polymer composite nanoparticles. They observed that the possibility of particle breakage was reduced with increased polymer solution viscosity, explaining the increased drug encapsulations at higher polymer concentrations.

A significant interaction between the molar concentration of FeCL_3_ and the surface modifier type on EE% was revealed, where increasing FeCL_3_ molar concentration (from 1 to 2 M) resulted in higher increase in the % EE in presence of CS as a surface modifier than that in the presence of CO. This might be explained on the basis of the higher stability of particles at high FeCL_3_ concentration in presence of CS which was confirmed by the high ZP as well. In addition, the ability of CS to form a very strong gel (Sacco et al., 2018), which could enhance the EE% by preventing DCN leakage from the particles.

**FIGURE 1:**
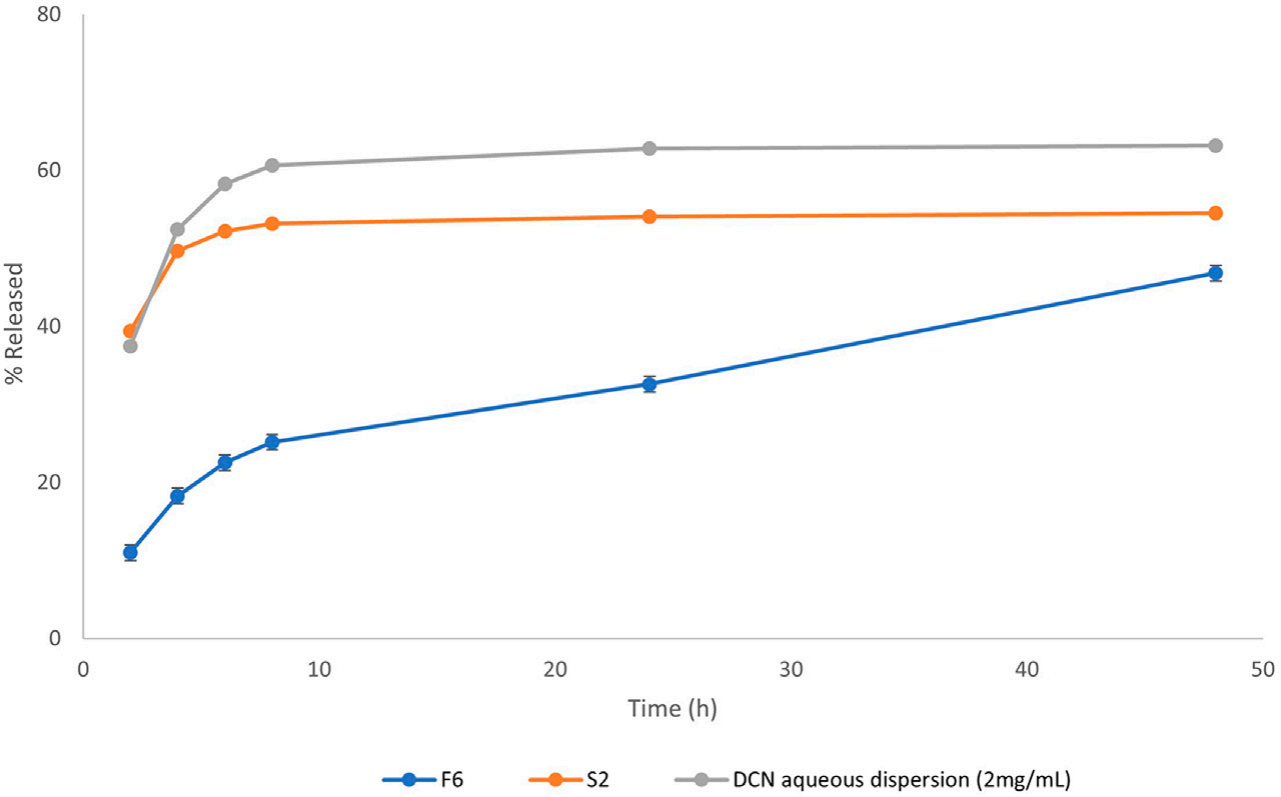
Release profiles of DCN from the optimized DCN-loaded magnetic particles formulation (F6) and DCN aqueous dispersion (2 mg/mL).

Regarding interaction between the concentrations of FeCl_3_ and surface modifier, it was found that increasing FeCL_3_ concentration (from 1 to 2M) at high surface modifier concentration (1%) resulted in higher increase in the EE% compared to that observed at low surface modifier concentration (0.5%). This might be attributed to that the higher concentration of both surface modifier and FeCL_3_ resulted in synergistic stabilization of particles, thus prevent the DCN from leakage (Franconetti et al., 2019), while at low surface modifier concentration particles were less stable and the tendency of DCN leakage was higher. In addition, it was observed that lower surface modifier concentration resulted in lower % EE in presence of CO than that in the presence of CS as a surface modifier, Figure 1F.

#### 3.2.2 Impact of variables on PS

As per ANOVA analysis, PS of the prepared DCN-loaded SMIOMPs was significantly affected by all the tested variables at 95% level of significance; the effects are illustrated as Supplementary Figure S2. However, no significant interaction was observed between variables. It was found that increasing molar concentration of FeCL_3_ from (1–2 M) decreases the molar ratio of Fe^2+^ to Fe^3+^ ions from 1 to 0.5 and thus decreases the PS (*p* = 0.0066). This may be attributed to the fact that when Fe^2+^ to Fe^3+^ ions molar ratio was greater than 0.5, the crystallites of Fe_3_O_4_ nanoparticles grew up slowly using excess Fe (OH)_2_ as per Schikorr reaction resulting in particles aggregation with consequent increase in PS (Shipko and Douglas, 1956; Mizutani et al., 2008; Jiang et al., 2011).

In addition, using CS as surface modifier resulted in smaller PS compared to that when using CO as surface modifier (*p* < 0.0001). This may be attributed to the denser coat formed by CS. Similar results were obtained by Frank et al. (2020) who observed that using CS as surface modifier for nanoparticles have high coating efficiency and thus lead to dense electrically stable coated particles with small particle and tends to avoid aggregation.

Regarding the surface modifier concentration, inverse relationship was observed with PS (*p* < 0.0001). This may be attributed to increasing the charge intensity by increasing the surface modifier concentration which lead to repulsion and deaggregation of particles leading to smaller PS (Frank et al., 2020). Similarly, Üstündaǧ-Okur et al. (2014) concluded that particles with high ZP, are electrically stable and tends to avoid aggregation, thus having small PS.

#### 3.2.3 Impact of variables on PDI

ANOVA analysis revealed a significant effect for only the surface modifier type on the PDI (*P*< 0.05). In addition, the surface modifier type exhibited a significant interaction with the concentration of either FeCL_3_ or surface modifier at the same significance level. Such significant effects and interactions are presented as Supplementary Figure S3. It was evident that CS surface-modified magnetic particles showed significantly lower PDI than CO surface-modified ones (*p* = 0.0019). This finding is in agreement with previous studies, where CS-coated nanoparticles achieved relatively lower PDI (≈0.34) then CO-based nanoparticles (≈0.77) (Cardoso et al., 2014; Wahyuni et al., 2021).

A significant interaction between the surface modifier type and either FeCL_3_ concentration or surface modifier concentration on the PDI was observed (*p* = 0.0002 and 0.0013), respectively. Raising the FeCL_3_ concentration (from 1 to 2M) in the prepared DCN-loaded IOMPs surface modified with CS resulted in increasing the PDI, whereas it resulted in decreasing the PDI in those surfaces modified with CO. This may be attributed to the higher ZP of CS surface modified particles which resulted in higher repulsive forces between particles with high FeCL_3_ concentration leading to high PS variation, while CO coated ones have lower ZP and less PS variation. Further, Changing the type of surface modifier from CS to CO at high surface modifier concentration (1%) resulted in increasing the PDI whereas it resulted in slight decreasing in the PDI at low concentration of surface modifier (0.5%). This may be due to the better coating efficiency of CS compared to that of CO at high concentration which led to smaller PS variation (Frank et al., 2020). On the other hand, at low concentration, the repulsive force between CS surface modified particles due to CS positive charge leads to greater PS variation than in the CO surface modified ones.

#### 3.2.4 Impact of variables on ZP

ANOVA results revealed that the absolute ZP of the DCN-loaded SMIOMPs is significantly affected by the three tested variables (*p* < 0.05). In addition, all the binary interactions between the tested variables were also significant at the same level of significance. The main effects and interactions between variables on absolute ZP are presented as Supplementary Figure S4. It was evident that higher FeCL_3_ molar concentration led to increased absolute ZP of the prepared DCN-loaded SMIOMPs (*p* < 0.0001). This could be credited to the fact that Fe^2+^: Fe^3+^ ion ratio of 0.5 tends to form more stable Fe_2_O_3_ than the higher ratio. Similarly, in their study on iron oxide nanoparticles, Alangari et al. (2022) reported higher positive charge on the surfaces of the developed Fe_2_O_3_ particles; they explained the high positive ZP due to using ammonia solution in the particles preparation and formation of positively charged Fe-OH_2_^+^ during Fe_2_O_3_ preparation.

The CS surface modified magnetic particles showed higher absolute ZP than those, whose surfaces are modified with CO (*p* < 0.0001) owing to higher charge density of CS compared to that of CO. Regarding the surface modifier concentration, a positive effect was observed on absolute ZP (*p* < 0.0001). This might be due to increasing the charge density with increasing the surface modifier concentration resulting in increasing the absolute ZP value of the prepared particles (Alangari et al., 2022).

A significant interaction between the molar concentration of FeCL_3_ and the surface modifier type on the absolute ZP values of the prepared DCN-loaded SMIOMPs was observed (*p* < 0.0001). Increasing FeCL_3_ molar concentration (from 1 to 2 M) resulted in higher absolute ZP in presence of CO than that in the presence of CS as a surface modifier. A significant synergistic interaction between the FeCL_3_ concentration (from 1 to 2M) and the surface modifier concentration on ZP was also observed (*p* < 0.0001). This might be attributed to the fact that charge density increases as the concentration of both FeCL_3,_ and surface modifier increases. Further, a significant interaction between surface modifier concentration and type on the absolute ZP values of the prepared DCN-loaded SMIOMPs was detected (*p* < 0.0001). Low concentration of surface modifier (0.5%) resulted in higher absolute ZP values when using CS as a surface modifier compared to CO, while at high concentration of surface modifier (1%), there was no marked difference between CS and CO modified particles. This could be credited to the fact that CO is negatively charged (Alangari et al., 2022), thus may bind the positively charged iron oxide particles and decrease the overall charge intensity. On the other hand, CS is positively charged, thus may increase the overall charge intensity of the positively charged iron oxide particles.

### 3.3 Statistical optimization

The optimized magnetic particles that could yield the highest EE%, lowest PS, and highest absolute ZP was chosen using Design expert^®^7. DCN-loaded SMIOMPs formulation (F6) that was prepared using 2 M FeCl_3_ and coated with 1% CS was the optimized system with the greatest desirability (0.951) according to the set optimization goals. The predicted (88% for EE%, 1.84 µm for PS, and 42 mV for ZP) and actual responses of the prepared optimized SMIOMPs (F6), presented in Table 1, were closely related to each other. The relatively low difference between estimated and predicted results confirmed the reliability of the design in estimating the optimal formulation.

### 3.4 *In-vitro* characterization of the optimized DCN-loaded SMIOMPs

#### 3.4.1 *In vitro* release

Release profile of the optimized DCN-loaded SMIOMPs (F6) compared to IOMPs (S2), and DCN aqueous dispersion is graphically presented in Figure 1. The optimized system (F6) was able to prolong DCN release compared to that from DCN-loaded IOMPs without surface modification (S2) and from DCN aqueous dispersion as well. Less than 50% of the drug was released throughout 48 h from the optimized DCN-SMIOMPs (F6), while about 50% of the drug was released during the first 4 h by the aqueous DCN dispersion and DCN-loaded IOMPs (S2). This demonstrates how crucial surface modification is for managing DCN release from the prepared SMIOMPs. Ebadi et al. (2021) achieved similar results in their study on the preparation of 5-fluorouracil loaded magnetic particles coated with polyvinyl alcohol. Their results revealed that the drug is located in the core and consequently, has to travel through a complex pathway, resulting in slow drug release.

#### 3.4.2 Shape and morphology

**FIGURE 2:**
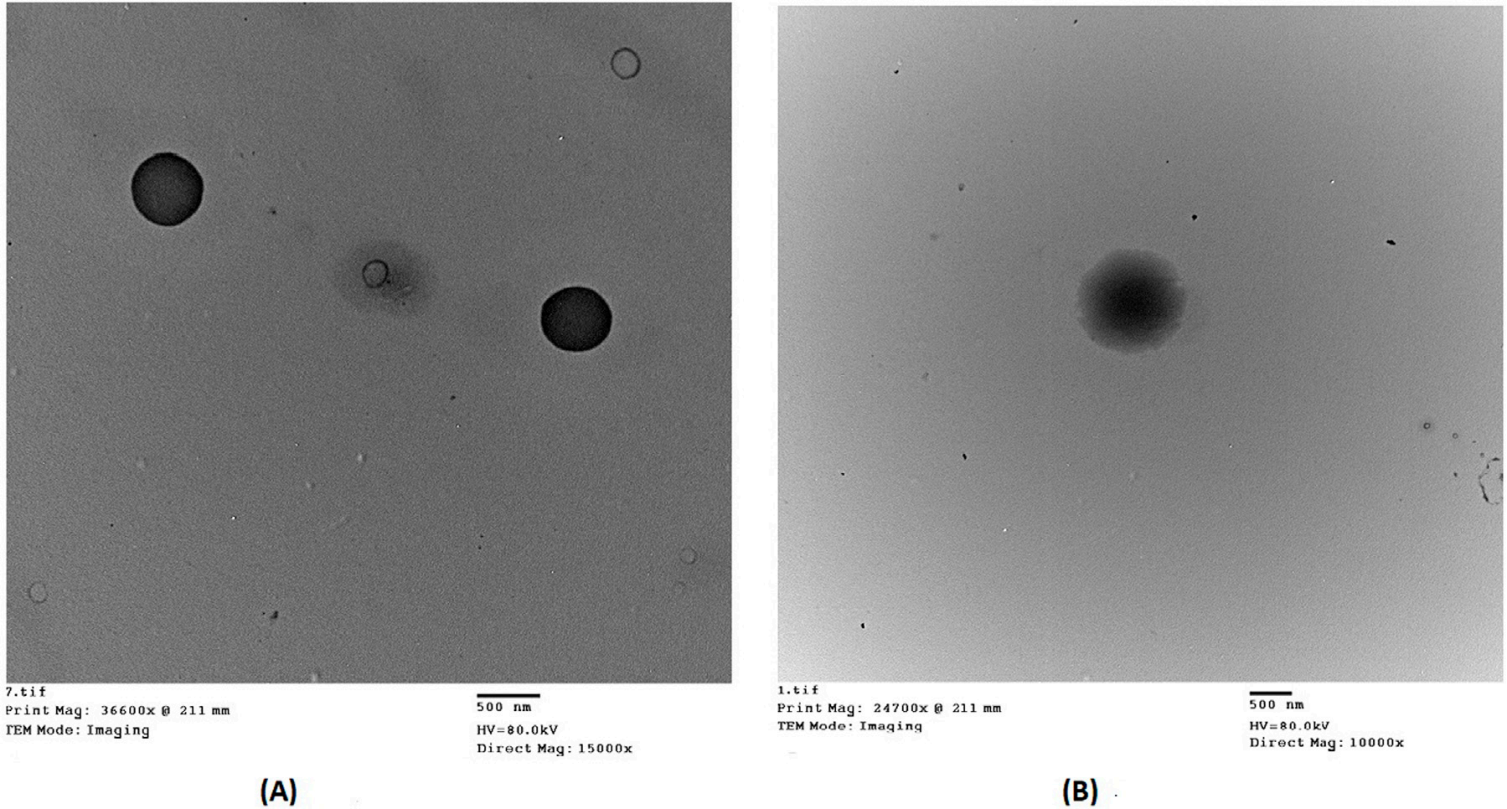
Transmission electron micrographs of: **(A)** DCN-loaded IOMPs (without CS); **(B)** optimized DCN-loaded SMIOMPs (with CS surface modification).

The optimized DCN-loaded SMIOMPs and DCN-loaded IOMPs (without surface modification with CS) TEM micrographs revealed non-aggregating particles with spherical shape, particle appeared to have smooth surface and sharp boundaries as shown in Figure 2A. Furthermore, TEM micrographs showed that the observed diameter was in agreement with the recorded size. Figure 2B revealed the surface modification with CS in the outer surface of the prepared optimized DCN-loaded SMIOMPs. Similar results were obtained by by Taherian et al. (2021) who described chitosan coated magnetic nanoparticles for breast cancer management.

### 3.5 Fourier-transform infrared spectroscopy (FTIR)

**FIGURE 3:**
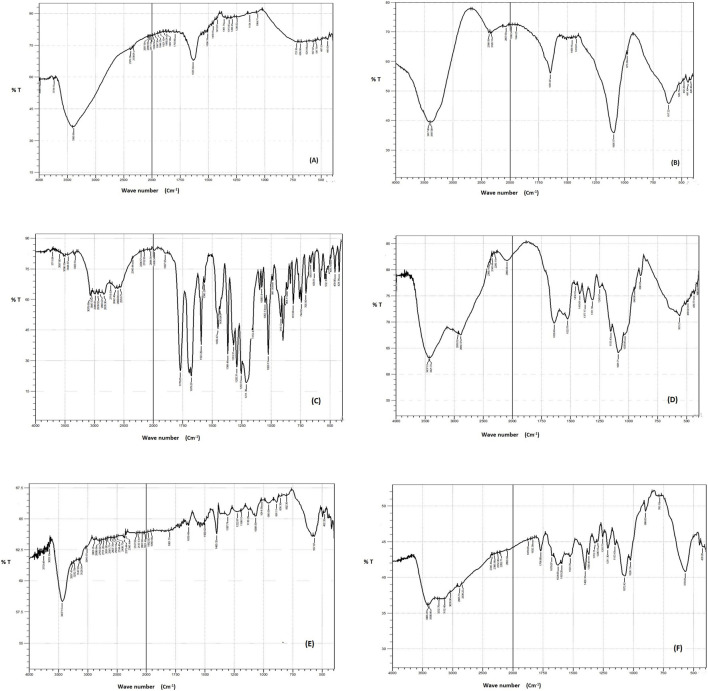
FTIR spectra of **(A)** FeCl_3_, **(B)** FeSO_4_, **(C)** DCN, **(D)** CS, **(E)** blank SMIOMPs, and **(F)** optimized SIOMPs F6. **(C)** is adopted from our previous work (Eladawy et al., 2021).

FTIR spectra were obtained for the individual components (DCN, FeCl_3_, FeSO_4_, and CS) as well as the blank SMIOMPs and the optimized lyophilized DCN-loaded SMIOMPs (F6), Figure 3. The IR chart of FeCl_3_, Figure 3A, showed bands near to 1,633 and 1739 cm^−1^, assigned to water molecules deformation, referring to the associated physiosorbed water on the surface of FeCl_3_. The band at 3249 cm^−1^ corresponding to the strong stretching vibrations of OH (Inam et al., 2018). The IR chart of FeSO_4_, Figure 3B, revealed sharp bands at 1,100 and 610 cm^-1^ which indicating the existence of SO_4_^2-^ ions. The adsorbed water molecules’ bending vibrations match the characteristic band at 1,640 cm^-1^. The broad absorption band between 3200 and 3500 cm^-1^ corresponds to the OH group presence (Ursescu et al., 2009). DCN IR spectrum, Figure 3C (Eladawy et al., 2021), exhibits distinctive bands at 3329 cm^−1^ assigned to the–OH stretching, 3070 cm^−1^ assigned to the aromatic C–H stretching, 2,939 cm^−1^ assigned to the aliphatic C–H stretching, 1770 cm^−1^ and 1,678 cm^−1^ assigned to the ester and carboxylic carbonyl groups, respectively, 1,593.20 cm^−1^ assigned to the aromatic C=C stretching, 705.96 cm^−1^ and 744.52 cm^−1^ assigned to the benzene and m-substituted benzene, respectively. The IR chart of CS, Figure 3D, revealed distinctive bands of polysaccharides in the fingerprint region from 1,156 to 890 cm^−1^ as that assigned to C–H on rings, C–O of alcohols, and C–O–C asymmetric band of glycoside bonds, also bands about 3300 cm^−1^, assigned to the hydrogen bonds of hydroxyl groups, and peaks at 1,633 and 1,523 cm^-1^ assigned to C=O and N–H bonds, respectively (Ataide et al., 2019). The IR chart of blank SMIOMPs, Figure 3E, showed the characteristic peaks for Fe_2_O_3_ particles and CS. The large broad band at 3398 cm^-1^ is assigned to the O-H stretching vibration in hydroxyl groups. The absorption bands around 1,635 cm^-1^, 1,508 cm^-1^ are due to the C=O asymmetric and symmetric bending vibration. The strong band below 700 cm^-1^ corresponds to Fe-O stretching mode. The band assigned to Fe-O stretching of Fe_2_O_3_ is seen at 567 cm^-1^ (Farahmandjou and Soflaee, 2015). The FTIR spectrum for the optimized DCN-loaded SMIOMPs system (F6), Figure 3F, shows the disappearance of the band 1770 cm^−1^ (corresponding to the stretching of C=O group of DCN carobxylic) as it could bind the amino group of CS (Taherian et al., 2021). There is not any undesired shift in the bands of both DCN and the single formulation components in the IR chart. Thus, based on FTIR spectra it can be concluded that the absence of any undesired chemical interaction between DCN and the components of the formulation.

### 3.6 Results of differential scanning calorimetry (DSC)

**FIGURE 4:**
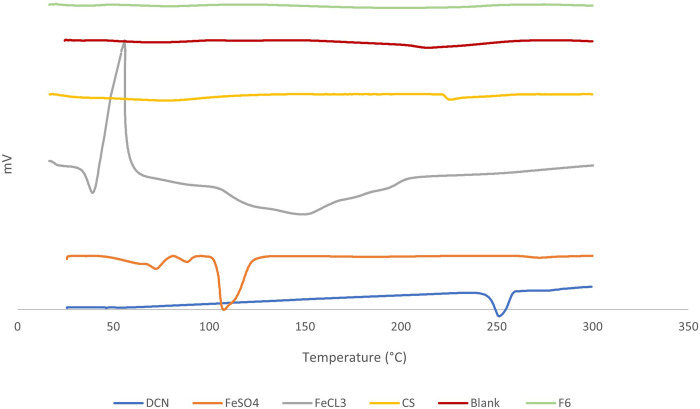
DSC thermograms of DCN, FeSO4, FeCl3, CS, blank SMIOMPs, and optimized SIOMPs F6. *DCN thermogram is adopted from our previous work* (Eladawy et al., 2021).

DSC was used as a common tool to explore the melting and recrystallization behavior of the formulation and its individual components (Balestrieri et al., 1996). Figure 4 shows the DSC thermograms for DCN, FeCl_3_, FeSO_4_, CS, as well as the blank IOMPs and the optimized lyophilized DCN-loaded SMIOMPs (F6). The DCN DSC thermogram revealed a sharp endothermic peak around 255 °C representing its melting temperature (Eladawy et al., 2021; Garthe et al., 2013). The DSC thermogram of FeCl_3_ showed an FeCl3 had an obvious exothermic peak appeared at about 65°C (Liu et al., 2016). The DSC thermogram of FeSO_4_ heptahydrate revealed three endothermic peaks at 71.46, 88.95, 118.44°C. The first peak at 71.46 °C corresponds to the melting point of the compound. The second peak at 88.95 °C could be credited to the dehydration of two water molecules from FeSO4 •6H_2_O to FeSO_4_ •4H_2_O, while the third one at 118.44°C might be due to the removal of 3 molecules of water from FeSO_4_ •4H_2_O to FeSO_4_ •H_2_O (Trivedi and Jana, 2019). The DSC thermogram of the CS exhibited an endothermic peak at 227.4°C originating from the melting of CS with the collapse of its structure (Ignjatović et al., 2018).

The blank IOMPs thermogram did not show any new peaks indicating the absence of any physical interactions; however, reduction in peaks intensity was observed due to dilution. DCN peak total disappearance was observed in the thermogram of the optimized lyophilized DCN-loaded SMIOMPs (F6) indicating that presence of DCN in the amorphous state within the iron oxide microparticles or its molecular dispersion in the microparticles. Similar results were reported in previous studies (Eladawy et al., 2021).

### 3.7 Effect of sterilization on the stability of the optimized DCN-loaded SMIOMPs formulation

In order to evaluate the effect of the sterilization process on the optimized formulation, re-evaluation was done after sterilization. Results revealed that process of sterilization had no marked impact on the properties of the prepared DCN-loaded SMIOMPs, where there was no significant change in the drug EE%, PS, and ZP (*p* > 0.05), Table 2, proofing the stability of the SMIOMPs upon exposure to gamma sterilization under the specified conditions.

**TABLE 2:** Characterization of the optimized DCN-loaded SMIOMPs (F6) before and after gamma sterilization.

	EE^a^ (%)	PS^a^ (µm)	ZP^a^ (mv)
Before sterilization	85.25 ± 0.25	1.56 ± 0.07	41.35 ± 0.7
After sterilization	86.00 ± 0.25	1.90 ± 0.02	41.0 ± 0.1

^a^ Data are mean values (n = 3) ± SD.

### 3.8 *In-vivo* study

**FIGURE 5:**
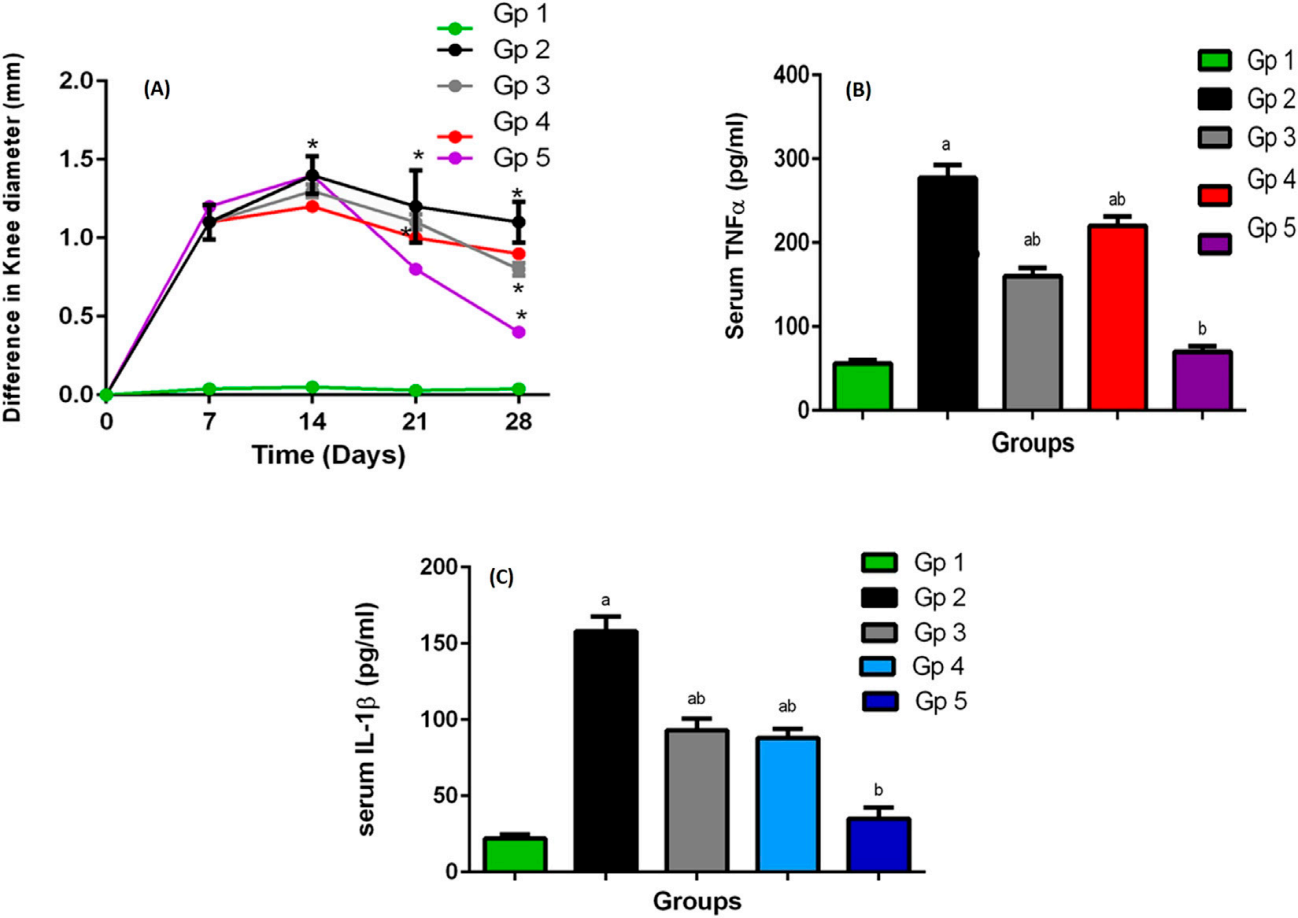
**(A)** The knee swelling for GP1 (negative control), GP2 (positive control), GP3 (Blank SMIOMPs), GP4 (DCN aqueous dispersion), and GP5 (optimized DCN loaded SMIOMPs (F6)), **(B)** The rat’s plasma TNF-α levels, **(C)** The rat’s plasma IL-1b levels. a Significantly different from negative control group at *p* < 0.05, * or b Significantly different from positive control group at *p* < 0.05, ab Significantly different from both negative and positive control group at *p* < 0.05.

The swelling of the knee of rats was estimated by determining their diameter, as an indicator of arthritis progression. The difference between the values that were recorded at normal condition and after arthritis induction showed the swelling of the knee at different times, Figure 5A. In comparison to the negative control, all the groups showed significant joint swelling at 95% significance level, at days 7 and 14, respectively (following 1 and 2 weeks of antigen injection). On day 21, 1 week apart from the treatment beginning, a significant reduction in the knee swelling was recorded at the same level of significance. Furthermore, the knee swelling through all the treated groups was significantly lower than the positive control group (*p* < 0.05).

At day 28, 1 week apart from receiving the treatment second dose, the treatment anti-edematous activity was clearly revealed, where the knee swelling of the groups was significantly reduced in the treated groups compared to the untreated ones; moreover, the difference among the treated groups became clearer. Results showed that the knee swelling degree followed the order: group 5 (treated with optimized DCN-loaded SMIOMPs (F6)) < group 4 (treated with DCN aqueous dispersion) < group 3 (treated with blank SMIOMPs) *p* < 0.05. The results were in accordance with the likely good effect of the selected formulation (F6).

Plasma interleukin 1-beta (IL-1b) and tumor necrosis factor-alpha (TNF-α) that represent the main proinflammatory cytokines, were significantly increased in the affected joint having large contribution to OA pathogenesis. Further, induction of other inflammatory mediators production, such as prostaglandins and cycloxygenase-2 from synovial cells was done by these cytokines leading to more cartilage inflammation and deterioration (Stannus et al., 2010; Kany et al., 2019). TNF-α and IL-1b serum levels, greatly elevated in such inflammatory condition, could be decreased by the correct utilization of anti-inflammatory agents; accordingly, they are regarded as appropriate markers for evaluation of synovial inflammatory disorders and pharmacological activity of actives (Larsson et al., 2015). Results of the plasma levels of TNF-α and IL-1b in rats measured following 4 weeks of OA induction are graphically illustrated in Figures 5B,C. The levels of TNF-α and IL-1b in the arthritic group (group 2) were elevated significantly (277 pg/mL for TNF-α and 158 pg/mL for IL-1b) relative to their levels in the treated groups (groups 3–5). A great correlation between TNF-α and IL-1b plasma levels and the knee swelling results was observed; the levels of such inflammatory markers followed the same order as the knee swelling degree. The superiority of the optimized DCN-loaded SMIOMPs (F6) was confirmed via achieving lowest serum levels (70 pg/mL for TNF-α and 45 pg/mL for IL-1b) over both blank SMIOMPs and the aqueous dispersion of DCN. The magnetic behavior of the iron oxide microparticles could be a major contributing factor to the observed superior performance. Such behavior allows for the direction of the particles by the external magnetic field enabling targeted delivery of DCN directly to the affected knee joint. This facilitates localized treatment while minimizing side effects (Tursunkulov et al., 2013). In addition, the controlled and prolonged release of the dug through the magnetic field could enhance the therapeutic efficacy with consequent improved outcomes (Hagit et al., 2010). It is worthy to note that localization of the iron oxide magnetic microparticles in the affected joint could generate localized heat when exposed to an alternating magnetic field; the developed magnetic hyperthermia could effectively aid in promoting cartilage regeneration and reducing inflammation through cellular and molecular signaling pathways (Abenojar et al., 2016; Guan et al., 2024).

### 3.9 Histopathological evaluation

**FIGURE 6:**
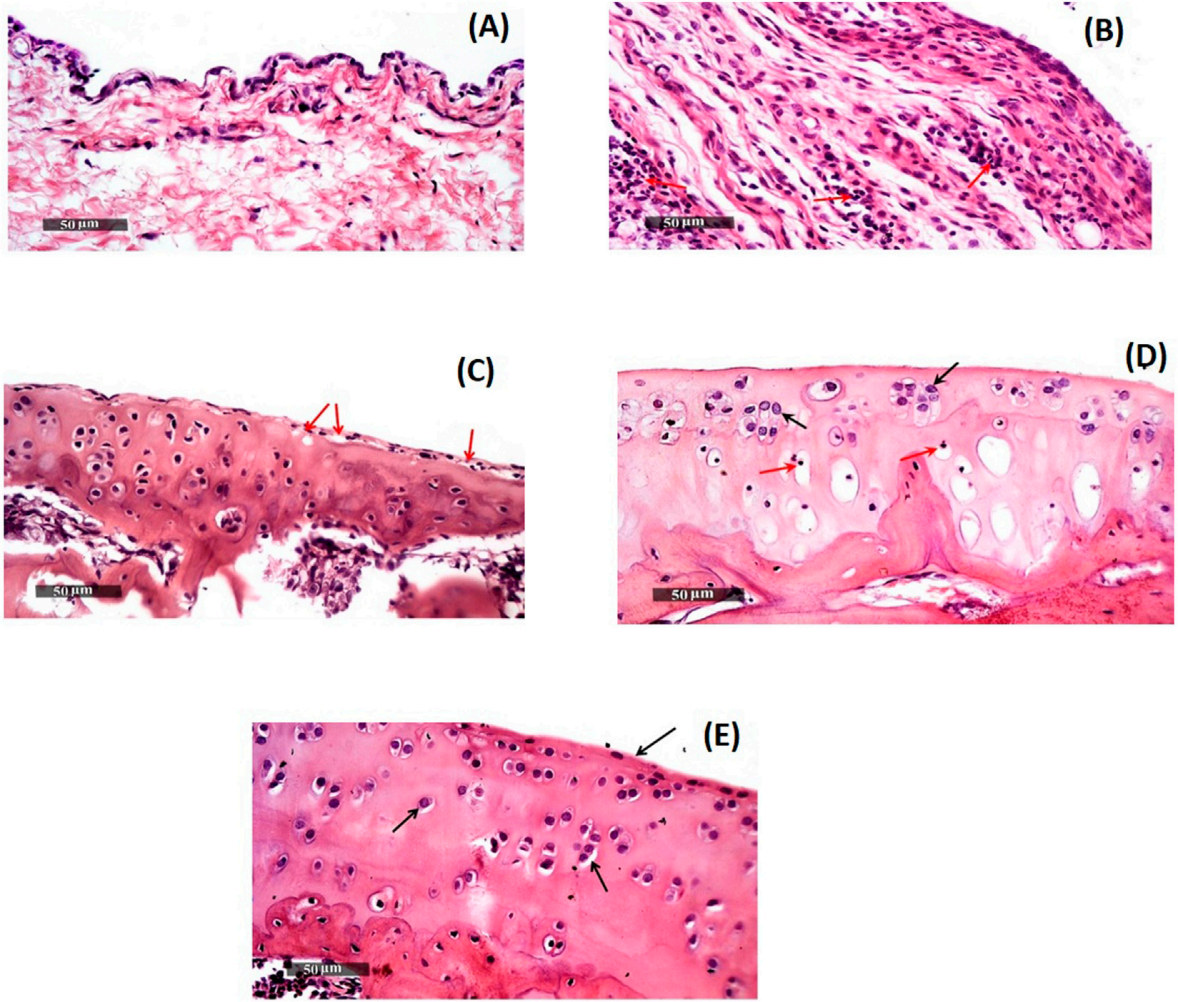
Morphological features of **(A)** GP.1 negative control, **(B)**GP.2 positive control, **(C)** GP.3 Blank SMIOMPs, **(D)** DCN aqueous dispersion, and **(E)** GP.5 Optimized DCN-loaded SMIOMPs (F6). Figure 6D is adopted from our previous work (Eladawy et al., 2021).

Modified-Mankin score system was used to assess the tibiofemoral articular cartilage degree of inflammation, Table 3. Inflammation was assigned to a score system starting from 0 to 14 according to modified Mankin grading ranging from 0 (minimum score) to 14 (maximal score) referring to normal cartilage and osteoarthritic model, respectively. Group 2 representing the positive control group had the highest score of 10, while group 5 (receiving DCN-loaded SMIOMPs) had the lowest score of 3.5. In addition, assessment of microscopic histopathological changes was done by using H&E stains, Figure 6.

**TABLE 3:** Modified Mankin grading for histopathological assessment.

Group	Modified mankin score^a^
Group 1 (-ve control)	0.5 ± 0.1
Group 2 (+ve control)	10.0 ± 1.0
Group 3 (Blank SMIOMPs)	7.5 ± 0.5
Group 4 (Drug 2 mg/mL)	4.0 ± 1.0
Group 5 (F6 DCN loaded SMIOMPs)	3.5 ± 0.5

^a^ Data are mean values (n = 6) ± SD.

Group 1 (negative control, mean Mankin score of 0.5) samples showed normal histological features of cartilages covered with hyaline and had intact smooth articular surfaces, all over cartilage zones with large vesicular intact nuclei and well organized apparent intact chondrocytes (Arrows). Uninjured synovial membranes were noticed with normal vasculatures and minimal records of infiltrates of inflammatory cells, Figure 6A.

Group 2 (positive control, mean Mankin score of 10) samples revealed subchondral congested blood vessels (dashed arrow) together with significant reduction of chondrocytes and large areas of cartilaginous surface fissures and erosions (star) with many necrotic and degenerated changes (red arrow). Many infiltrates of inflammatory cells in synovial membranes (red arrow, Figure 6B) with covering epithelium showing slight focal hyperplasia.

Regarding, Group 3 (receiving SMIOMPs, mean Mankin score of 7.5), 50% of samples showed focal areas of separation and fissures of superficial articular cartilage (red arrow) with mild mononuclear inflammatory cells infiltrates in synovial membranes, Figure 6C.

Samples of group 4 (rats IA injected with 0.5 mL of the DCN aqueous dispersion, mean Mankin score of 4) samples, Figure 6D (Eladawy et al., 2021), showed relatively smooth, intact articular surfaces without any changes (black arrow) with blood vessels congestion (star). Synovial membranes revealed few scattered infiltrates of inflammatory cells (red arrow) with degenerative changes appeared as focal areas and deeper cartilaginous zones showed pyknotic nuclei (red arrow).

Group 5 (receiving optimized SMIOMPs F6, mean Mankin score of 3.5) showed well organized apparent intact histological features, normal cartilages covered with hyaline and chondrocytes showing large vesicular intact nuclei (Arrows), and the smooth articular surfaces were also intact. Samples demonstrated intact synovial membranes with normal vasculatures and minimal infiltrates of inflammatory cells, Figure 6E.

These results showed that DCN-loaded SMIOMPs has the ability to improve the knee swelling results, in addition to reducing plasma levels of proinflammatory mediators, demonstrating the marked chondrogenic impact of the optimized formulation. Such findings suggest that DCN-loaded SMIOMPs could promote marked healing effect in case of OA inflammation via suppressing inflammation, as well as promoting the repair of tissues by virtue of the advantages of the magnetic iron oxide microparticles as previously discussed.

## 4 Conclusion

In the current study, intra-articular injectable SMIOMPs loaded with DCN were successfully formulated via using iron salts, and surface modified with natural polymer (CS or CO). Full factorial design was successfully employed for optimizing the proposed formulation. Statistical analysis revealed that using 2 M FeCL_3_ and 1 M FeSO_4_ in presence of 33% ammonia solution led to the production of SMIOMPs with the minimized size, maximized absolute ZP and EE% with overall desirability of 0.951. Further *in vivo* study showed that the optimized formulation obviously improved the swelling of rats’ knees. Therefore, this DCN-loaded SMIOMPs could be considered as an appropriate alternative for DCN oral treatment.

## Data Availability

The original contributions presented in the study are included in the article/Supplementary Material, further inquiries can be directed to the corresponding author.

## Supplementary material

SUPPLEMENTARY FIGURE S1 Line charts for the effect of **(A)** FeCL_3_ concentration, **(B)** surface modifier type, and **(C)** surface modifier and interaction plots for the interaction between **(D)** FeCL_3_ molar concentration and surface modifier type, **(E)** FeCL_3_ molar concentration and surface modifier concentration, and **(F)** surface modifier concentration and surface modifier type on the EE%.

SUPPLEMENTARY FIGURE S2 Line charts for the effect of **(A)** FeCL_3_ concentration, **(B)** surface modifier type, and **(C)** surface modifier on PS.

SUPPLEMENTARY FIGURE S3 Line chart **(A)** for the effect of surface modifier type and interaction plots for the interaction between **(B)** FeCL_3_ molar concentration and surface modifier type and **(C)** surface modifier concentration and surface modifier type on the PDI.

SUPPLEMENTARY FIGURE S4 Line charts for the effect of **(A)** FeCL_3_ concentration, **(B)** surface modifier type, and **(C)** surface modifier and interaction plots for the interaction between **(D)** FeCL_3_ molar concentration and surface modifier type, **(E)** FeCL_3_ molar concentration and surface modifier concentration, and **(F)** surface modifier concentration and surface modifier type on the absolute ZP.
