# Supplementary figures and images for: Diacerein-loaded surface modified iron oxide microparticles (SMIOMPs): an emerging magnetic system for management of osteoarthritis via intra-articular injection
Source: Front Bioeng Biotechnol. 2024 Oct 28;12:1439085. doi: 10.3389/fbioe.2024.1439085 (PMC11551035; doi:10.3389/fbioe.2024.1439085)

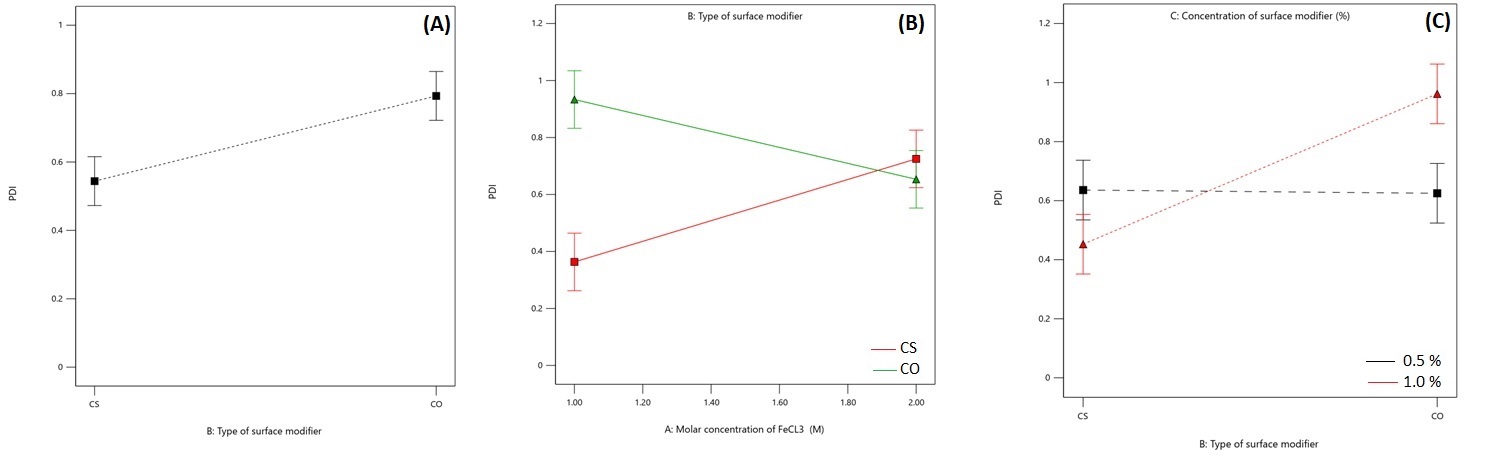

Supplement: Supplementary file 1 [file Image3.JPEG]

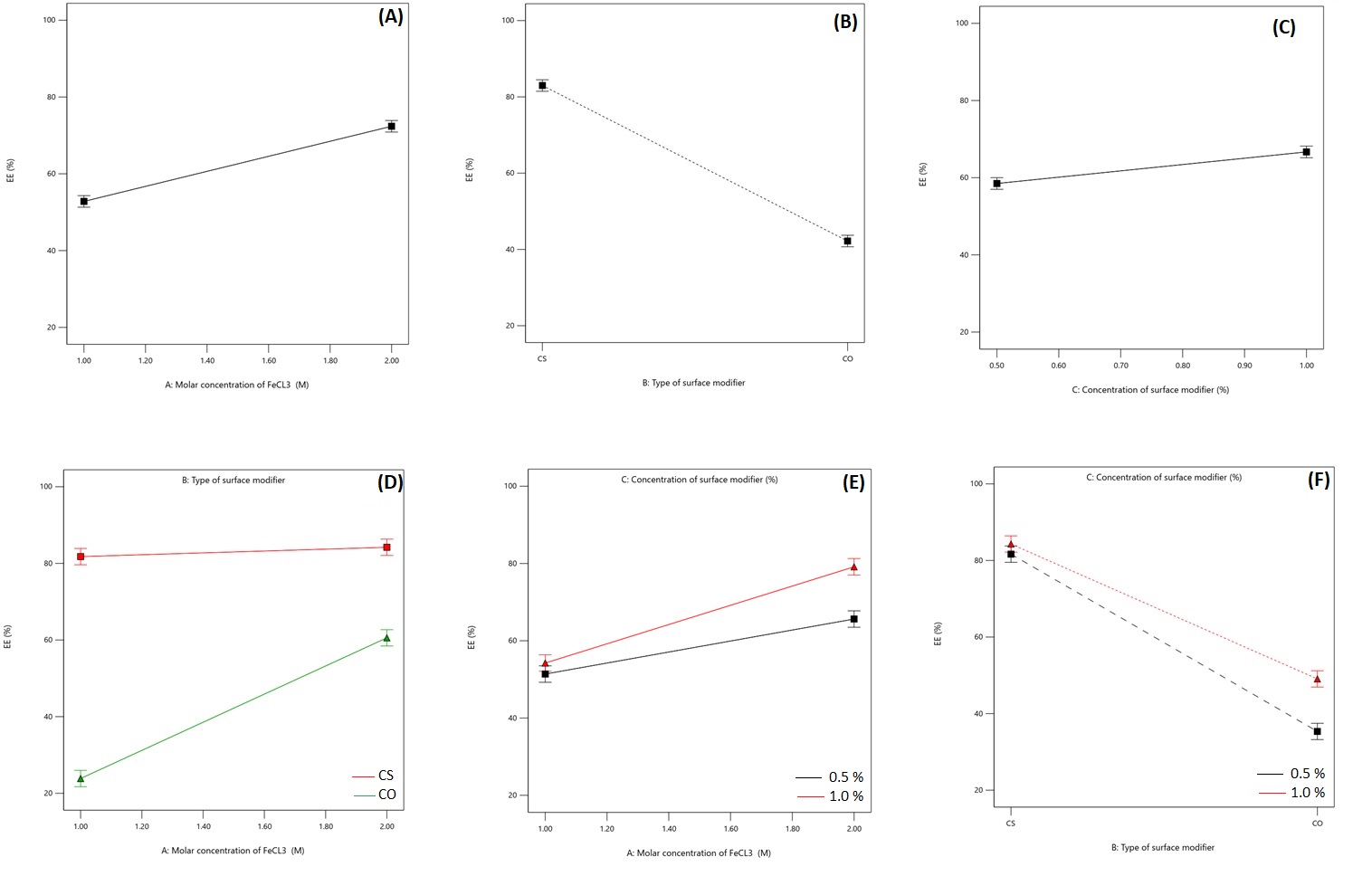

Supplement: Supplementary file 2 [file Image1.JPEG]

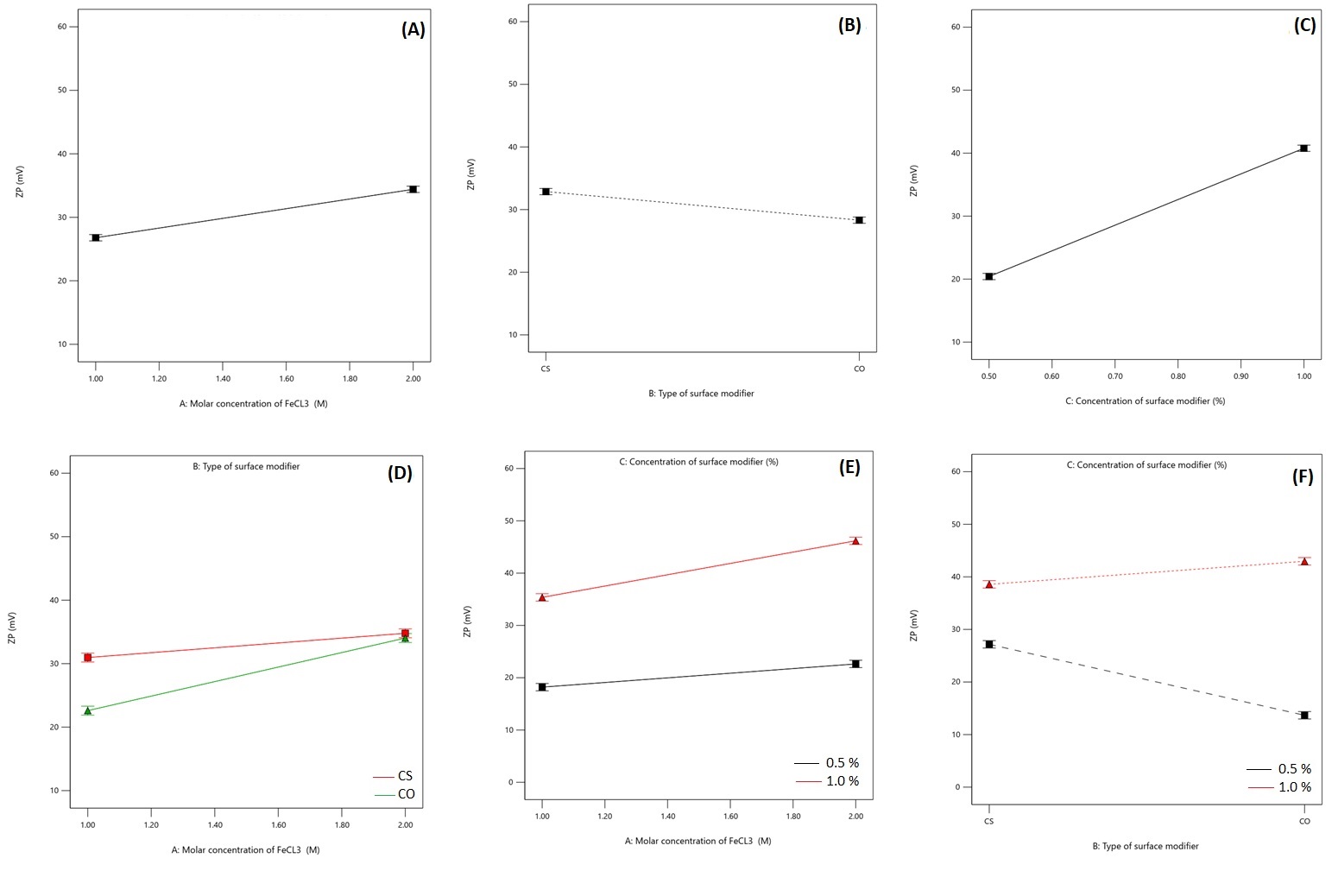

Supplement: Supplementary file 3 [file Image4.JPEG]

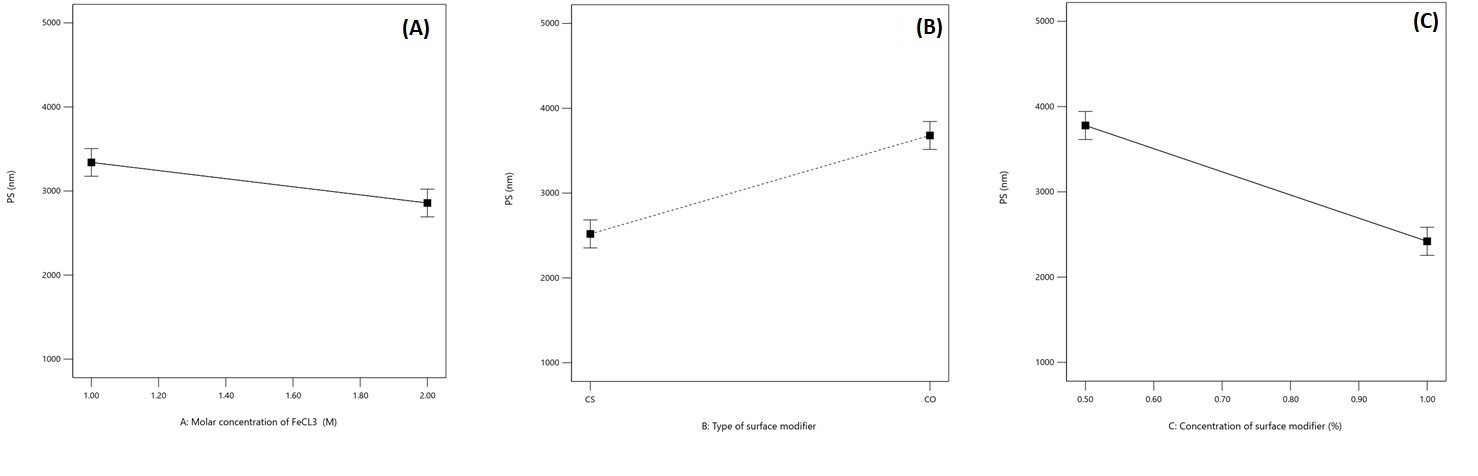

Supplement: Supplementary file 4 [file Image2.JPEG]
